# Nuclear Translocation of LDHA Promotes the Catabolism of BCAAs to Sustain GBM Cell Proliferation through the TxN Antioxidant Pathway

**DOI:** 10.3390/ijms24119365

**Published:** 2023-05-27

**Authors:** Zhujun Li, Zhiyan Gu, Lan Wang, Yun Guan, Yingying Lyu, Jialong Zhang, Yin Wang, Xin Wang, Ji Xiong, Ying Liu

**Affiliations:** 1 Department of Pathology, School of Basic Medical Sciences, Fudan University, Yixueyuan Rd. 138, Shanghai 200032, China; 18211010050@fudan.edu.cn (Z.L.); 22211010064@m.fudan.edu.cn (Z.G.); 20211010059@fudan.edu.cn (L.W.); 18301050183@fudan.edu.cn (J.Z.); 2 Cyberknife Center, Department of Neurosurgery, Huashan Hospital, Fudan University, 12 Middle Wulumuqi Road, Shanghai 200040, China; yguan10@fudan.edu.cn (Y.G.); wangxinck@fudan.edu.cn (X.W.); 3 Department of Neurosurgery, Huashan Hospital, Fudan University, 12 Middle Wulumuqi Road, Shanghai 200040, China; 19111220075@fudan.edu.cn; 4 Department of Oncology, Institutes of Biomedical Sciences, Shanghai Medical College, Fudan University, Shanghai 200032, China; 5 Department of Pathology, Huashan Hospital, Fudan University, 12 Middle Wulumuqi Road, Shanghai 200040, China; yinwang88@hotmail.com

**Keywords:** lactate dehydrogenase A, glutamate, branched-chain amino acid transaminase 1, redox balance, thioredoxin, GBM, *IDH*-wild type

## Abstract

Glutamate is excitotoxic to neurons. The entry of glutamine or glutamate from the blood into the brain is limited. To overcome this, branched-chain amino acids (BCAAs) catabolism replenishes the glutamate in brain cells. Branched-chain amino acid transaminase 1 (BCAT1) activity is silenced by epigenetic methylation in *IDH* mutant gliomas. However, glioblastomas (GBMs) express wild type *IDH*. Here, we investigated how oxidative stress promotes BCAAs’ metabolism to maintain intracellular redox balance and, consequently, the rapid progression of GBMs. We found that reactive oxygen species (ROS) accumulation promoted the nuclear translocation of lactate dehydrogenase A (LDHA), which triggered DOT1L (disruptor of telomeric silencing 1-like)-mediated histone H3K79 hypermethylation and enhanced BCAA catabolism in GBM cells. Glutamate derived from BCAAs catabolism participates in antioxidant thioredoxin (TxN) production. The inhibition of BCAT1 decreased the tumorigenicity of GBM cells in orthotopically transplanted nude mice, and prolonged their survival time. In GBM samples, BCAT1 expression was negatively correlated with the overall survival time (OS) of patients. These findings highlight the role of the non-canonical enzyme activity of LDHA on BCAT1 expression, which links the two major metabolic pathways in GBMs. Glutamate produced by the catabolism of BCAAs was involved in complementary antioxidant TxN synthesis to balance the redox state in tumor cells and promote the progression of GBMs.

## 1. Introduction

ROS are byproducts of numerous cellular processes. A key hallmark of cancer cells is rapid and unrestricted growth. Therefore, cancer cells sustain a much higher level of ROS production than normal cells. Elevated ROS are generally detrimental to cells, and the expression of various antioxidants in cancer cells needs to be upregulated, which can buffer ROS and promote the progression of the tumor. Glutamate plays a vital role in the redox balance of cells [1]. Glutathione (GSH) is the major antioxidant, and is synthesized from glutamate. Harris reported that, with the growth of a tumor, thioredoxin (TxN) acts synergistically with GSH to buffer ROS levels, and the synthesis of TxN requires the export of glutamate in exchange for the import of cysteine [2]. Glutamate is a non-essential amino acid that can be obtained from food. Most cells in the body can directly import glutamate from the blood (20~50 µM/L) [3]. In the brain, glutamate depolarizes neurons and acts as the primary excitatory neurotransmitter, but its import from the blood into the brain does not occur to any appreciable extent [4]. BCAAs are generally leucine, valine, and isoleucine. The BCAAs are swiftly transported into the brain. The brain possesses abundant BCAT1, which catabolizes BCAAs to replenish glutamate. Yudkoff found that BCAAs contributed to at least one-third of the amino groups utilized to form glutamate [5]. In the brain, astrocytes are the cells for the synthesis of glutamate derived from the metabolism of BCAAs. Astrocytoma, a tumor originating from abnormally proliferating astrocytes, can also metabolize BCAAs to acquire a large amount of glutamate [6].

Tönjes reported that BCAT1 protein expression was absent in *IDH*-mutated gliomas [7]. *IDH* mutation can result in abnormal catalytic activities, catalyzing α-ketoglutarate (α-KG) to produce the cancer metabolite 2-hydroxyglutarate (2-HG) [8]. Accumulated 2-HG competes with intracellular α-KG-dependent dioxygenases, including DNA demethylases, resulting in DNA hypermethylation [9]. McBrayer found that in *IDH* mutant gliomas, the expression of the BCAT1 was significantly inhibited due to 2-HG-mediated hypermethylation that resulted in a significant decrease in the production of intracellular glutamate from BCAAs. This partially explains why a better prognosis is observed in *IDH* mutant gliomas than those without mutations [10]. All these studies confirmed the critical role of BCAA catabolism in sustaining the intracellular glutamate pool in glioma cells. However, the metabolic regulation of BCAAs and their roles in maintaining the redox balance of GBMs have not been studied yet.

LDHA is the key enzyme catalyzing the production of lactic acid from pyruvate in glycolysis. LDHA is highly expressed in many malignant tumors, as well as in GBMs [11]. In the cervical epithelium, Liu reported that after HPV infection, ROS accumulated in cervical epithelial cells, which resulted in the entry of LDHA into the nucleus and the acquisition of non-classical enzymatic activities, which further activated the DOT1L-mediated epigenetic modification of H3K79 hypermethylation [12]. BCAT1 is one of the targets of DOT1L [13]. However, it has yet to be studied whether LDHA also enters the nucleus, facilitating BCAA metabolism under oxidative stress in glioma cells.

In the present study, we analyzed the data of 549 GBM cases in TCGA and found that BCAT1 was positively correlated with the expression of LDHA and DOT1L. Treating GBM cells with H_2_O_2_ caused ROS accumulation to promote the entry of LDHA into the nucleus. In GBM cells, nuclear LDHA activates DOT1L, which mediates the H3K79 hypermethylation of the *BCAT1* gene, resulting in elevated BCAT1 expression. *BCAT1* knockdown, achieved by shRNA, inhibited the proliferation, invasion, and migration of GBM cells, accompanied by downregulated intracellular antioxidant TxN production. More importantly, the supplementation of *BCAT1* knockdown cells with glutamate remarkably recovered the level of TxN, suggesting that BCAA metabolism is vital for TxN synthesis in GBM cells. In addition, a high level of BCAT1 was correlated with poor prognosis in GBM patients. Together, our study verified that the nuclear localization of LDHA in GBMs triggered by ROS accumulation resulted in activated BCAAs catabolism to maintain the intracellular redox balance and promote cell proliferation, which favored the progression of GBMs.

## 2. Results

### 2.1. ROS Induce Nuclear Translocation of LDHA in GBM Cells

**Figure 1:**
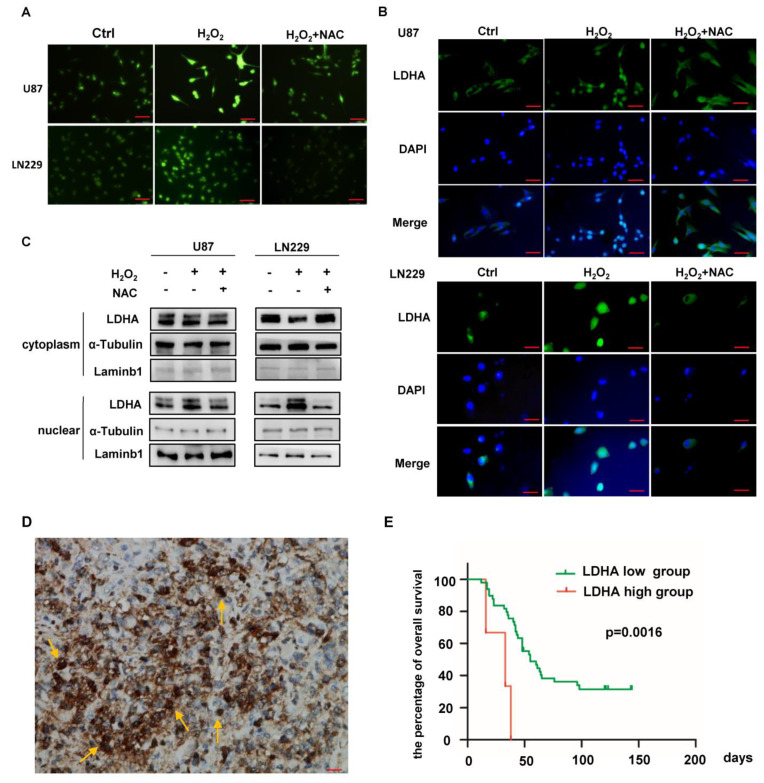
ROS promotes the nuclear translocation of LDHA in GBM cells. (**A**) GBM cells were treated with H_2_O_2_ and supplemented with NAC, as indicated. Cellular ROS were measured by DCFH-DA. (**B**) Immunofluorescent images of LDHA in GBM cells. (**C**) Nuclear was isolated from GBM cells, followed by blotting with LDHA, Tubulin, and Lamin B1. The results are representative of three independent experiments. (**D**) Representative IHC images showing LDHA expression in GBM samples. Arrows indicated LDHA nuclear-positive cells. (**E**) Overall survival curve of GBM patients with a high and a low level of LDHA (log-rank test, *p* = 0.0016). Scale bars, 100 μm. All data are shown as the mean ± SEM. Values of *p* < 0.05 were considered statistically significant.

Liu reported that LDHA is preferably located in the nucleus in cervical tumors due to HPV-induced intracellular ROS accumulation [12]. Cancer cells have a much higher level of ROS production than normal cells due to their rapid proliferation, so we first treated U87 and LN229 cells with hydrogen peroxide (H_2_O_2_) to induce ROS accumulation and supplemented them with N-acetyl-L-cysteine (NAC), a ROS scavenger, to eliminate ROS (Figure 1A). We found that LDHA rapidly translocated from the cytoplasm to the nucleus after H_2_O_2_ treatment, and the nuclear translocation of LDHA was eliminated with NAC supplementation (Figure 1B). Then, we performed nuclear and cytoplasmic isolation assays and found a similar pattern for LDHA localization (Figure 1C). We also found HIF-1α induced in hypoxia cultured glioma cells, and the expressions of LDHA and BCAT1 were also increased (Supplementary Figure S1). This finding indicates that in GBM cells, the nuclear translocation of LDHA is dependent on ROS. To further validate this, we performed LDHA IHC staining in GBM samples. Thirty-nine of fifty-five GBM samples (70.91%) were LDHA-positive, and all harbored LDHA-nuclear translocation tumor cells (Figure 1D). The level of LDHA expression was significantly associated with a poor prognosis in cancer patients (Figure 1E).

### 2.2. Nuclear LDHA-Induced BCAT1 Expression in GBM Cells in a DOT1L Activity-Dependent Manner

**Figure 2:**
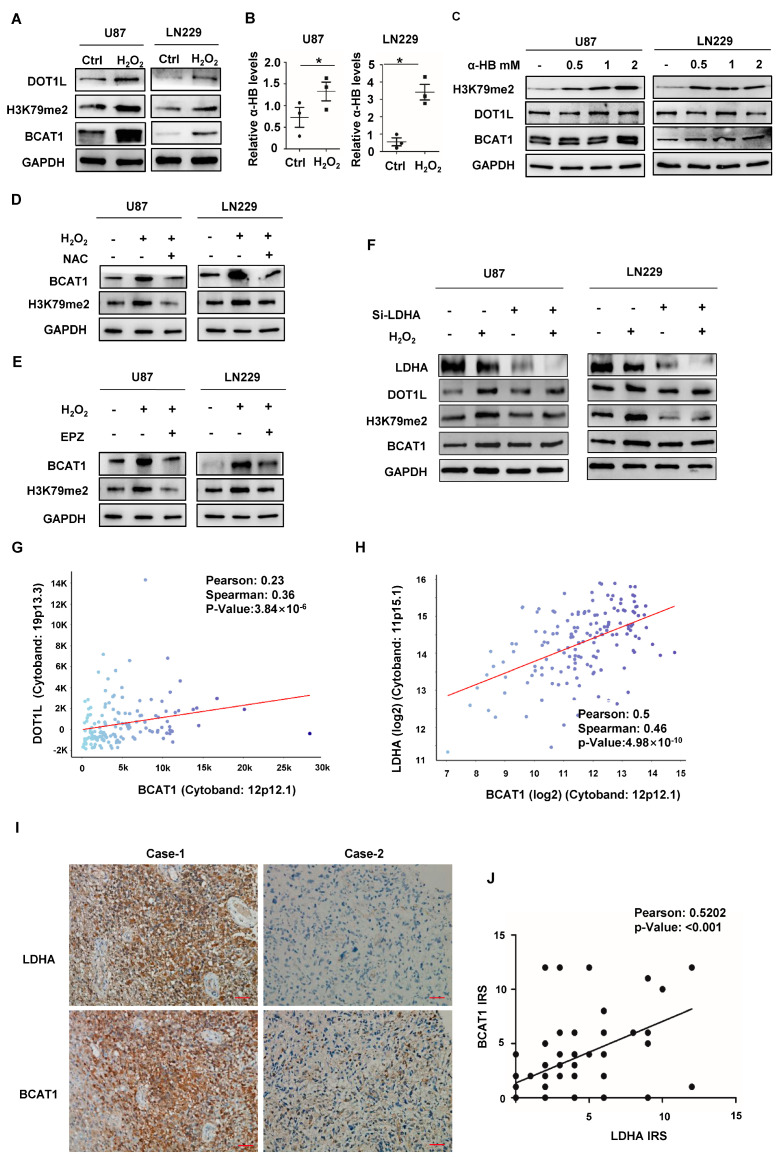
DOT1L mediates LDHA-induced BCAT1 expression in PGBM cells under ROS accumulation. (**A**) GBM cells were treated with H_2_O_2_ and supplemented with NAC for blotting DOT1L, H3K79me2, and BCAT1, as indicated. (**B**) The α-HB from GBM cells was analyzed with LC-MS/MS, and the relative abundance (by metabolite peak area) was normalized to cell numbers. (**C**) H3K79 methylation levels and the expression of DOT1L and BCAT1 were analyzed in GBM cells upon α-HB treatment for 24 h. Ctrl, control. (**D**) H_2_O_2_ promotes BCAT1 expression and H3K79 methylation level in GBM cells, which are diminished by NAC. GAPDH was used as a loading control. (**E**) DOT1L mediates BCAT1 expression. GAPDH was used as a loading control. (**F**) *LDHA* knockdown by siRNA significantly inhibited the expression of BCAT1 after H_2_O_2_ treatment in GBM cells. (**G**,**H**) Positive correlations were found between the mRNA levels of *LDHA* and *BCAT1* (Pearson correlation γ was 0.5202; *p* < 0.01) and *DOT1L* and *BCAT1* (Pearson correlation γ was 0.5202; *p* < 0.01) in 549 TCGA GBM samples. (**I**) Representative IHC images for BCAT1 and LDHA in GBM samples. In case 1, both BCAT1 and LDHA were positive. In case 2, the expression of LDHA was negative, and BCAT1 was weakly positive. Scale bar, 100 μm. (**J**) A positive correlation was found between the IRS of LDHA and BCAT1 (Pearson correlation γ is 0.5202; *p* < 0.01). The results are from three independent experiments. IRS: immunostaining scores. All data are shown as the mean ± SEM. The *p* values were determined by a two-tailed *t*-test. Values of *p* < 0.05 were considered statistically significant. * denotes *p* < 0.05. Scale bar, 100 μm.

Aberrant metabolic alterations have been confirmed to widely participate in epigenetic and epitranscriptomic modifications [14]. We found that levels of H3K79 hypermethylation, DOT1L, and BCAT1 in GBM cells were increased upon H_2_O_2_ treatment (Figure 2A). In LC-MS/MS, we found that the concentration of α-HB was significantly increased in GBM cells treated with H_2_O_2_ (Figure 2B). Notably, α-HB caused a significant upregulation in the levels of H3K79 Hypermethylation, DOT1L, and BCAT1 (Figure 2C). Then, we found that NAC or EPZ004777 (a DOT1L specific inhibitor) markedly recovered the H_2_O_2_-induced expression of H3K79 hypermethylation and BCAT1 (Figure 2D,E), suggesting that ROS accumulation and activated DOT1L were essential for H3K79 hypermethylation and BCAT1 expression. H3K79 hypermethylation and the increased expressions of DOT1L and BCAT1 after H_2_O_2_ treatment were eliminated after LDHA knockdown (Figure 2F). The positive correlations of DOT1L with BCAT1 (Pearson correlation γ was 0.5202; *p* < 0.01) and LDHA with BCAT1 (Pearson correlation γ was 0.5202; *p* < 0.01) were analyzed from gene expression profiles of 549 GBM cases of the Cancer Genome Atlas (TCGA) database, respectively (Figure 2G,H). In CGGA (Chinese Glioma Genome Atlas), a significant positive correlation was found between LDHA and BCAT1 expression in both primary and recurrent gliomas, and a similar positive correlation was observed in gliomas at all malignant levels (Supplementary Figure S2). In IHC staining (representative images shown in Figure 2I), we also found a positive correlation (Pearson correlation γ was 0.5202; *p* < 0.01) between the IRS of LDHA and BCAT1 (Figure 2J). All these data indicate that nuclear LDHA increases BCAT1 expression in GBM cells.

### 2.3. BCAT1 Expression Is Critical for Sustaining GBM Cell Proliferation

**Figure 3:**
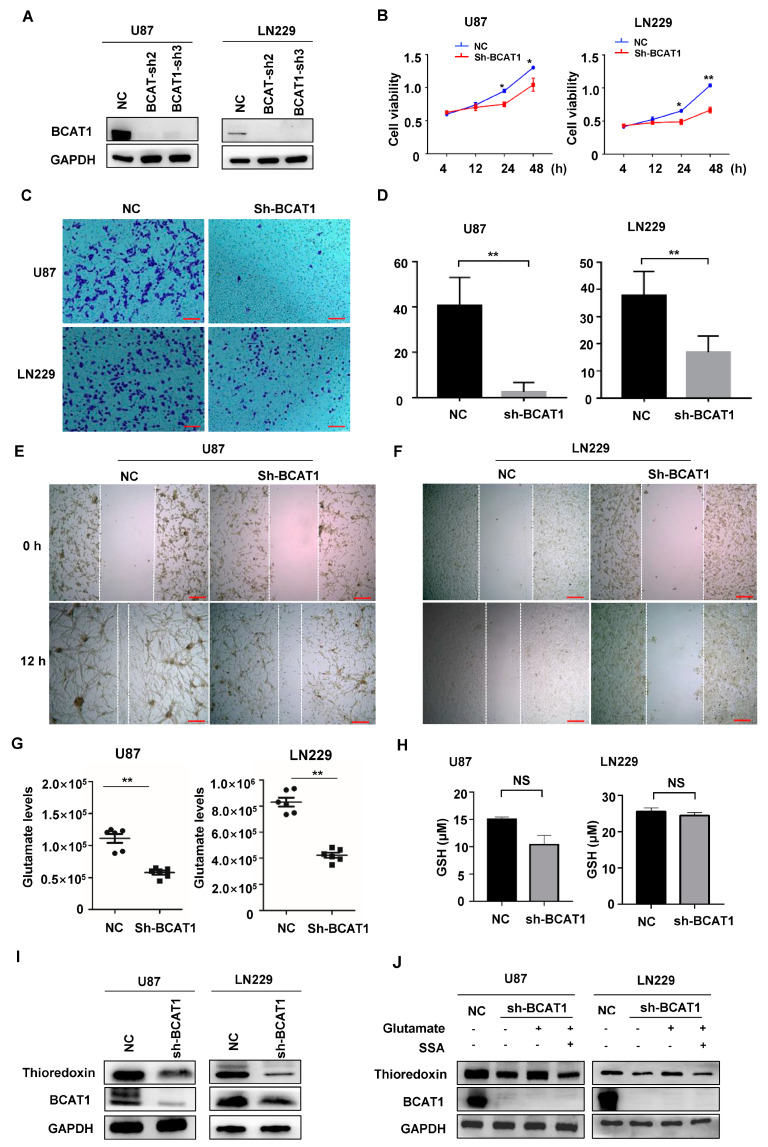
BCAT1 expression is correlated with GBM cell viability, migration, and invasion. (**A**) *BCAT1* expression was inhibited by shRNA (*sh-BCAT1*) in GBM cells. Empty vector-transfected cells were used as a control (Vec). (**B**) CCK-8 assays indicated the viability of pGBM cells in the sh-BCAT1 or Vec group. (**C**,**D**) Representative images and statistical analysis of transwell assays of GBM cells in the *sh*-*BCAT1* or Vec group. (**E**,**F**) Representative images of wound healing assays in GBM cells at 0 and 12 h in the *sh*-*BCAT1* or Vec group. GBM cell migration significantly depended on BCAT1 expression. The results are representative of three independent experiments. (**G**) *BCAT1* knockdown decreased cellular glutamate. Glutamate from GBM cells was analyzed with LC–MS/MS, and the relative abundance (by metabolite peak area) was shown. Relative metabolite abundances were normalized to cell numbers. (**H**) Total glutathione levels in GBM cells. No difference was seen between the *sh-BCAT1* and Vec groups. (**I**) *BCAT1* knockdown by shRNA decreased TxN expression in GBM cells. (**J**) Glutamate supplementation replenished TxN expression in *BCAT1* knockdown GBM cells. GAPDH was used as a loading control. All data are shown as the mean ± SEM. Significance was assessed using Student *t*-tests and one-way analysis of variance. * *p* < 0.05, ** *p* < 0.01; NS, not significant. CCK-8: Cell Counting Kit-8. Scale bar, 100 μm.

BCAAs are essential nutrients and energy sources for cancer growth. BCAT1 is the first enzyme to initiate the catabolism of BCAAs. We found that silencing *BCAT1* with shRNA (Figure 3A) decreased the cell proliferation, migration, and invasion of GBM cells, as assessed with CCK-8 (Figure 3B), transwell assays (Figure 3C,D), and cell scratch (Figure 3E,F), respectively. To investigate whether BCAA catabolism is critical for glutamate production in GBM cells, we quantified the cellular glutamate concentration with LC-MS/MS and found that the inhibition of BCAT1 significantly decreased the level of glutamate in GBM cells (Figure 3G).

Next, we examined the expression of glutathione (GSH) and TxN, two glutamate-derived antioxidants in GBM cells, after *BCAT1* knockdown. The GSH detection assay showed no significant changes in GSH levels between *BCAT1* knockdown and wild-type cells (Figure 3H). However, TxN expression was decreased in *BCAT1*-deficient cells (Figure 3I). Cystine/glutamate transporter system Xc-, also known as SLC7A11, is responsible for the export of glutamate and the import of cystine. Consistently, we found that glutamate replenishment can significantly enhance the expression of TxN in GBM cells lacking BCAT1 expression, while the application of sulfasalazine (SSA), a chemical inhibitor of the Xc-transporter, inhibited the effect of glutamate replenishment (Figure 3J). These results indicate that glutamate derived from BCAA metabolism is essential for TxN synthesis in GBM cells.

### 2.4. BCAAs Sustain Malignant Progression of GBM Tumors

**Figure 4:**
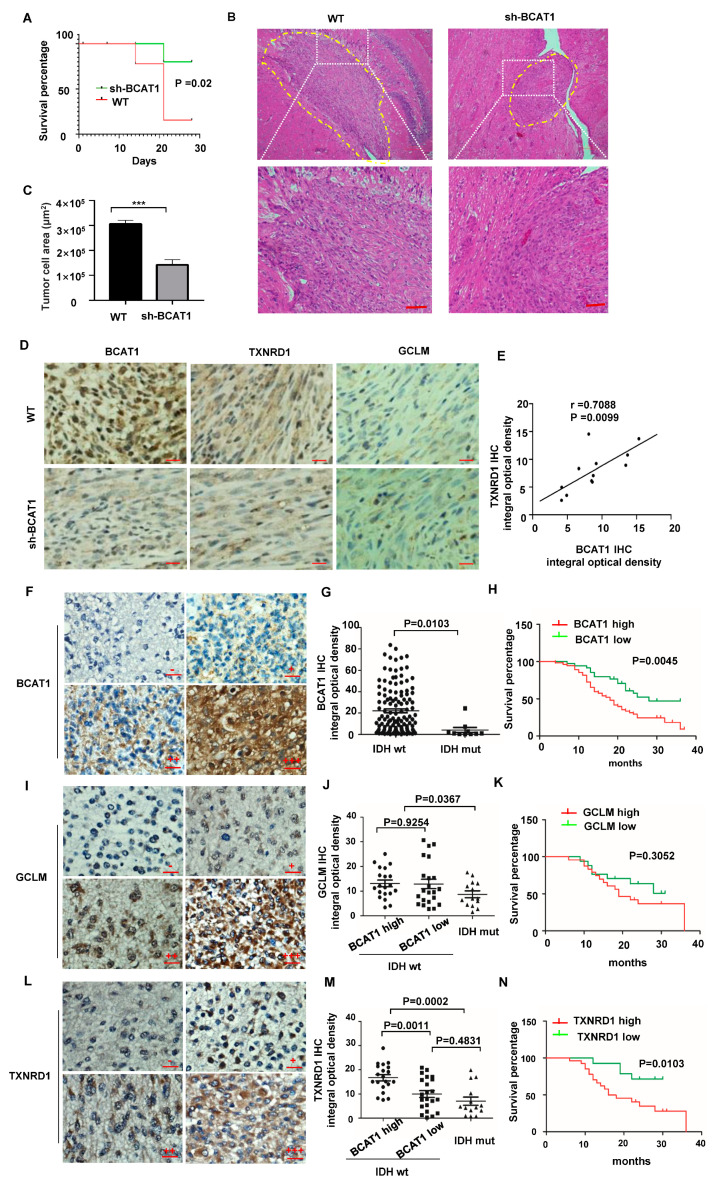
BCAAs sustain the malignant proliferation of GBM tumors. (**A**) The survival percentage of nude mice orthotopically transplanted with *BCAT1* wild-type and *BACT1*-deficient LN229 cells, respectively. There was a significant difference in the OS time between these two groups (*p* = 0.02). There were 16 biologically independent mice in each group. (**B**) Representative images of H&E staining of orthotopically transplanted tumors. (**C**) Quantification of tumor size of LN229 cells transplanted orthotopically in a nude mouse model. All data are shown as the mean ± SEM. Mean ± SEM, two-tailed T test, *p* =0.0003. (**D**) Representative images of IHC staining of BCAT1, GCLM, and TXNRD1 in orthotopically transplanted tumors. (**E**) Positive correlation between the staining density of BCAT1 and TXNRD1. (Pearson correlation: r = 0.7088, *p* =0.0099). (**F**) Representative images of BCAT1 staining in GBM samples. Negative (−), weakly positive (+), moderately positive (++), and strongly positive (+++). (**G**) Statistical analysis of BCAT1 IHC integral optical density in 90 GBM samples (*IDH* wild-type) and 14 diffuse astrocytoma samples (grade 4, *IDH* mutant). (**H**) Kaplan–Meier survival analysis showed that GBM patients with higher BCAT1 expression had shorter OS than those with lower BCAT1 expression (*p* = 0.045, log-rank test). (**I**) Representative images of GCLM staining in GBM samples. Negative (−), weakly positive (+), medium positive (++), and strong positive (+++). (**J**) Statistical analysis of GCLM IHC integral optical density in 90 GBM samples (*IDH* wild-type) and 14 diffuse astrocytoma samples (grade 4, *IDH* mutant). (**K**) Kaplan–Meier survival analysis showed that GCLM expression showed no correlation with OS in GBM samples (*p* = 0.3052, log-rank test). (**L**) Representative images of TXNRD1 staining in GBM samples. Negative (−), weakly positive (+), moderately positive (++), and strongly positive (+++). (**M**) Statistical analysis of TXNRD1 IHC integral optical density in 90 GBM samples (*IDH* wild-type) and 14 diffuse astrocytoma samples (grade 4, *IDH* mutant). In GBM samples, TXNRD1 was significantly higher in the high BCAT1 group than in the low BCAT1 group (*p* = 0.001). (**N**) Kaplan–Meier survival analysis showed that GBM patients with higher TXNRD1 expression had shorter OS than those with lower BCAT1 expression (*p* = 0.0103, log-rank test). All data are shown as the mean ± SEM. The *p* values were determined by a two-tailed *t*-test. Scale bar, 100 μm. *** *p* < 0.001.

We used a tumor orthotopic mouse model to test the role of BCAA catabolism in GBM cell growth. At day 28, a total of 11 out of 16 (68.75%) nude mice with *BCAT1* wild-type LN229 cells were dead, while only 6 of 16 (40%) nude mice with *BACT1*-deficient LN229 cells were dead (Figure 4A), showing a significant difference in the OS time between these two groups (*p* = 0.02). Tumor cells showed infiltration into the peripheral brain tissues in the *BCAT1* wild-type groups, whereas tumors had a sharp and clear border in the *BCAT1* knockdown group (Figure 4B). *BCAT1* knockdown significantly reduced the tumor size compared to the size of the tumors in the control group (Figure 4C). These findings indicate a moderate invasion in *BCAT1*-deficient cells. The rate-limiting step of GSH synthesis was carried out by glutamate-cysteine ligase (GCL), which is a heterodimer of catalytic (GCLC) and modifier (GCLM) subunits. In IHC staining, we found that the expression of GCLM showed no difference between the BCAT1 high and low expression groups (*p* = 0.56) (Figure 4D). However, we observed a decreased expression level of TXNRD1, the key enzyme of TxN synthesis, in the shBCAT1 group compared with that of the wild-type group (*p* = 0.0476). The expression of BCAT1 and TXNRD1 was positively correlated (γ = 0.7088; *p* = 0.0099) (Figure 4E).

BCAT1 expression is silent in *IDH* mutation glioma due to extensive DNA hypermethylation within the promoter region of BCAT1. Consistently, we found that BCAT1 expression was low in histologically characterized grade 4 (*IDH* mutant) diffuse astroglioma samples, but high in GBM samples (*IDH* wild-type), which was negatively correlated with patients’ overall survival times (OS) (Figure 4F–N). GCLM showed no correlation with BCAT1 levels and the patient’s OS time (Figure 4I–K), while TXNRD1 was positively correlated with BCAT1 expression and poor prognosis (Figure 4L–N). This result indicates that the alternative TxN antioxidant pathway fulfills the compensatory role of GBM progression.

This finding provides evidence that glutamate, the byproduct of BCAA catabolism, participates in redox homeostasis in GBM cells in addition to the roles of BCAAs, acting as an essential nutrient source to fuel tricarboxylic acid (TCA) cycle metabolism and to supply nitrogen for de novo nucleotide and amino acid synthesis.

## 3. Discussion

Accelerated glycolysis constitutes a critical aspect of cancer metabolism, as reported by Warburg in 1956 [15]. LDHA is NAD-dependent and facilitates the glycolytic process by catalyzing the transformation between pyruvate and lactate. In many tumors, the expression of LDHA is elevated, which is associated with malignant progression [16]. Enhanced LDHA is an independent poor prognostic factor of nasopharyngeal carcinoma, non-Hodgkin lymphoma, cervical cancer, and melanoma [11]. Liu reported that LDHA translocated into the nucleus in response to increased ROS driven by HPV16/18 infection in cervical epithelial cells [12]. We also found that LDHA was translocated to the nucleus in all GBM patients, and its increased expression was associated with a poor prognosis of these patients. In *IDH* wild-type GBM cells, LDHA nuclear translocation was induced by H_2_O_2_ and alleviated by NAC, which indicated that LDHA nuclear translocation was associated with ROS accumulation in GBMs. We also proved that nuclear LDHA triggered the DOT1L-mediated H3K79 methylation of BCAT1, resulting in increased BCAA catabolism in GBM cells. The nuclear translocation of LDHA has been significantly enhanced by ROS accumulation, and LDHA plays a role in modulating BCAA metabolism in cancer cells. Our study provides evidence that the enzyme responsible for glucose metabolism is involved in altering the catabolism of amino acids in tumor cells.

Metabolic changes in BCAA affect glioblastoma cell proliferation through multiple mechanisms [17]. The first step in BCAA catabolism is the transfer of an α-amino group to α-ketoglutarate (α-KG), yielding glutamate and the corresponding branched-chain α-ketoacids [18]. The latter were then catabolized to acetyl coenzyme A and succinyl-CoA and subsequently oxidized in the tricarboxylic acid cycle for ATP synthesis and macromolecule precursors for cell growth [19]. In this study, we found that ROS triggered the nuclear translocation of LDHA, which subsequently regulated BCAT1 expression; we focused on the role of BCAA catabolism in the redox balance in cells. Glutamate is the substrate for producing GSH, which is the most abundant antioxidant within cells [1]. In addition, glutamate is exported in exchange for cystine into the cells that can be reduced to cysteine in an NADPH-dependent manner to upregulate the alternative TxN antioxidant pathway [20]. Therefore, glutamate participates in both GSH and TxN synthesis. In the brain, glutamate is an excitatory neurotransmitter, which means that the import of glutamate from blood does not occur to any appreciable extent. BCAAs serve as an essential source of glutamate in the brain [21]. This means that cells and tumors in the brain depend heavily on BCAA catabolism to replenish glutamate. We verified that a high level of BCAT1 in GBMs predicted a poor prognosis. The expression of BCAT1 was positively correlated with the level of thioredoxin reductase 1 (TXNRD1), which is the key enzyme of the TxN antioxidant pathway. Therefore, BCAAs metabolism mainly affects the expression of TxN, but has no significant impact on the content of GSH, which may be explained by glutamate being directly involved in GSH synthesis, and TxN synthesis being an indirect effect of importing cysteine through the exchange of glutamate.

In cultured GBM cells and nude mice with orthotopic transplantation, *BCAT1* knockdown by shRNA significantly retarded the malignant proliferation of GBM cells. Both glycolysis and BCAA catabolism play roles in maintaining the rapid growth of tumor cells. The regulatory factors of these two pathways are likely to become the treatment target for tumors. The increased expression of BCAT1 in various malignant tumors has been verified [22]. Dietary depletion of BCAAs might be an effective way of halting tumor progression [23]. Inhibitors of LDHA, such as oxalate and gossypol, can cut off the ATP supply of tumor cells without affecting the direct utilization of pyruvate in normal cells [24]. Our research indicated that oxidative stress triggered the entry of LDHA into the nucleus, which manipulated BCAA metabolism to acquire supplementary glutamate for the synthesis of the antioxidant TxN to sustain glioma cell proliferation. This pathway might be unique in the brain, where glutamate cannot be imported from the blood due to the limitation of the blood–brain barrier. This approach might help to explore new strategies targeting the noncanonical activities of the LDHA enzyme or BCAAs diet control by harnessing the redox state of the GBM cells.

In conclusion, ROS accumulation in *IDH* wild-type GBM tumor cells leads to the entry of LDHA to the nucleus and promotes the metabolism of BCAAs. Glutamate produced by the catabolism of BCAAs is involved in the synthesis of TxN, which is conducive to maintain the redox state of tumor cells and to promote the progression of GBMs (shown schematically in the graphic abstract). Our research indicates that the internal modulation of LDHA by BCAT1 expression links two major metabolic pathways of BCAAs and aerobic glycolysis in GBMs. Here, we preliminarily explored the potential regulatory mechanism of elevated BCAAs catabolism in GBMs. Further study on the targeted inhibition of BACC catabolism and its effect on tumor growth will provide a new method for GBM treatment.

## 4. Materials and Methods

### 4.1. Antibodies and Reagents

Antibodies against LDHA (Cell Signaling Technology, Danvers, MA, USA, 3582, 1:2500), DOT1L (Cell Signaling Technology, 77087, 1:1000), H3K79me2 (Cell Signaling Technology, 5427, with 1:1000), BCAT1 (Cell Signaling Technology, Cat.88785, 1:1000), Tubulin (Santa Cruz, Santa Cruz, CA, USA, Cat. sc-5286, 1:2000), Lamin B1 (Cell Signaling Technology, Cat.13435, 1:2000), β-actin (Santa Cruz, Cat. sc-047778, 1:2000), and GAPDH (Santa Cruz, Cat.sc-47724, 1:2000) were used for Western blotting.

Antibodies against LDHA (Cell Signaling Technology, 3582, 1:500), BCAT1 (Cell Signaling Technology, Cat.88785, 1:500), GCLM (Proteintech, Rosemont, IL, USA, Cat.14241, 1:500), and TXNRD1 (Proteintech, Cat.11117, 1:500) were used for immunohistochemistry staining (IHC).

Hydrogen peroxide solution (H_2_O_2_) (Sigma, St. Louis, MO, USA, Cat.323381), N-acetyl cysteine (NAC) (Sigma, Cat.A7250), DAPI (Sigma, Cat.D9542), sodium 2-hydroxybutyrate (Santa Cruz, Cat. sc-258161), sulfasalazine (SSA) (Sigma, Cat.S0087), and EPZ004777 (Selleck, Washington, DC, USA, Cat.S7353) were commercially obtained.

### 4.2. Human GBM Samples

Archived, formalin-fixed, paraffin-embedded human GBM samples diagnosed by two neuropathologists in line with the World Health Organization (WHO) guidelines [25] were acquired from Huashan Hospital, Fudan University (Shanghai, China), between September 2010 and December 2011. The specimens included ninety GBMs (wild type *IDH*) and fourteen diffuse astrogliomas (*IDH* mutant with histological grade 4). The average age of patients was 52.08 ± 15.35 years, with a male to female ratio of 1.86:1 (male: 65 cases; female: 35 cases). All of the patients signed informed consent prior to this study. The Ethics Committee of Fudan University approved the procedures associated with the acquisition and usage of human astrocytoma samples (2019-Y004). All procedures for obtaining and handling the human tissue samples complied with the Helsinki Declaration (1964, amended most recently in 2013) [26].

### 4.3. Cell Culture

GBM cell lines U87 and LN229 were obtained from the American Type Culture Collection (ATCC). GBM cells were cultured in Dulbecco’s modified Eagle’s medium (DMEM) (Invitrogen, Carlsbad, CA, USA) supplemented with 10% fetal bovine serum (FBS) (Invitrogen), 100 U/mL penicillin G, 100 mg/mL streptomycin sulfate (Gibco-BRL Life Technologies, Gaithersburg, MD, USA), and 1% L-glutamine (Invitrogen).

### 4.4. Cell Treatment

For H_2_O_2_ treatment and NAC supplementation, GBM cells U87 and LN229 were plated in a complete culture medium with 10 μM H_2_O_2_ for 6 h and then exchanged in a medium supplemented with 1 mM NAC for another 6 h.

For glutamate and SSA treatment, cells were plated in a complete culture medium with 10 μM glutamate with or without 250 nM SSA for 24 h.

### 4.5. Generation of Stable Cell Pools

To generate stable BCAT1 knockdown GBM cells, small hairpin RNA (shRNA) targeting *BCAT1* was commercially obtained from Obio Tech. Corp., Ltd. Shanghai, China. The sequence of sh-BCAT1 was 5′-GCCGCATCTTGAGCAAATT-3′. GBM cells were infected with the retrovirus and selected with 2 μg/mL puromycin for 1 week.

LDHA siRNA was commercially acquired from Hanbio Ltd., Shanghai, China. The sequences of *LDHA* siRNA were F: 3′-UUCGAUGACAUCAACAAGATT-5′. R: 3′-UAAAGUACCCYGUGCUCAATT-5′.

### 4.6. Measurement of Intracellular ROS Levels

ROS levels were determined using an active oxygen detection kit (s0033, Biyuntian Biotechnology Ltd., Wuhan, China). Briefly, cells were incubated with 5 μM H2DCF-DA (chloromethyl-2′, 7′-dichlorofluorescein diacetate) for 30 min at 37 °C to load the fluorescent dye.

### 4.7. Nuclear Isolation

Isolation of nuclei was performed using a commercially available nuclei isolation kit (NUC201, Sigma Aldrich St. Louis, MO, USA). Briefly, GBM cells in the lysis medium were carefully placed on top of a 1.8 M sucrose gradient and centrifuged at 30,000× *g* for 45 min in a precooled swinging bucket ultracentrifuge. Nuclei were collected as a centrifuged pellet.

### 4.8. Liquid Chromatography-Mass Spectrometry Analysis

One million cells were collected and resuspended in ice-cold 80% methanol. Samples were vigorously vortexed and placed in liquid N2 to freeze. Samples were centrifuged at 13,000× *g* for 15 min. The supernatant was evaporated, and metabolomic analyses were performed as outlined previously. LC analysis was performed using a 1290 Infinity LC system (Agilent, CA, USA). Mass spectra acquisition was performed using a 5500 QTRAP mass spectrometer (AB SCIEX, Framingham, MA, USA) in the positive ionization mode and MRM mode [27]. The resulting metabolites were normalized based on protein concentration.

### 4.9. Cell Proliferation Assay

Ten thousand GBM cells were seeded on a 96-well plate and incubated in 0.2 mL of culture medium. The cell proliferation rate was determined using a Cell Counting Kit-8 (CCK-8) (Dojindo Laboratories, Tokyo, Japan) by following the manufacturer’s instructions.

### 4.10. Transwell Migration Assay

The Transwell migration assay was performed on GBM cells using a Boyden chamber containing a polycarbonate filter with a pore size of 8 μm (Corning Costar, Corning, NY, USA). Migrating cells were fixed in 4% paraformaldehyde and stained with crystal violet. Five random fields were selected, and the number of migrating cells was counted. All experiments were performed in triplicate.

### 4.11. Wound Healing Assay

GBM cells were seeded and cultured to 90% confluence before wounding with a 200 μL pipette tip. Pictures were taken at 0 h and 18 h after scratching.

### 4.12. Immunofluorescence and Microscopy

Cells were plated on glass coverslips and fixed with 4% paraformaldehyde. After permeabilization with 0.2% Triton-X-100, cells were incubated with the primary antibody overnight at 4 °C, then incubated with the secondary antibody for 4 h at 4 °C. After counterstaining with DAPI (5 mg/mL), the coverslips were coverslipped and imaged by an OLYMPUS IX81 Microscope.

### 4.13. Western-Blotting Analysis

For Western blot analysis, total proteins were extracted using the radioimmunoprecipitation assay buffer (RIPA) containing complete protease and phosphatase inhibitors (Selleck, Houston, TX, USA). The protein concentration was determined using the BCA Protein Assay (Cat No. A53226 Thermo Fisher Scientific, Waltham, MA, USA). The blots were incubated with primary antibodies followed by incubation with HRP-tagged secondary antibodies (Abcam, Cambridge, UK). Proteins of interest were visualized using an enhanced chemiluminescence kit (Cat No. 89880 Thermo Fisher Scientific, Waltham, MA, USA).

### 4.14. Orthotopic Transplantation

Animal experiments were carried out after receiving approval from the Institutional Animal Care and Use Committee (IACUC) of Fudan University (20180302-051). BALB/c nude mice (female, 8 weeks old) were purchased from Shanghai SIPPR-BK Laboratory Animal Company (Shanghai, China) and randomly divided into two groups (*BCAT1wt* and *shBCAT1*) with 16 mice in each group. Each mouse was stereotaxically injected with 6 µL (2 × 10^5^) of LN229 cells into the left striatum. These mice were monitored and euthanized when they demonstrated morbidity signs, including hunched posture, lethargy, difficulty in ambulating, and weight loss, or after one month. On day 28, all mice were sacrificed and their brains were dissected and cut conically to obtain the biggest segment of the tumor. This segment was then fixed in formalin, embedded in paraffin, and cut into sections for H&E staining and immunostaining.

Image J software (U.S. National Institutes of Health, http://rsb.info.nih.gov/ij/download.html accessed on 1 July 2021) with customized macros [28] was used to quantify tumor areas that were defined by two neuropathologists.

### 4.15. Immunohistochemistry (IHC) Staining

IHC staining procedures were performed as previously described [29]. In brief, 3% H_2_O_2_ was applied to quench endogenous peroxidases, followed by antigen retrieval using the citrate buffer. Primary antibodies were incubated overnight at 4 °C. Staining was performed using an LSAB+ kit (DAKO Cytomation, Glostrup, Denmark). 3,3-diaminobenzidine was used as a chromogen and Mayer’s hematoxylin was used for nuclear staining.

To semi-quantify the IHC staining, five fields (173 µm^2^ each) from each sample were randomly selected microscopically and images were captured using a charge-coupled device (CCD) camera. The IMT i-Solution software version 10.1 (IMT i-Solution, Inc., Burnaby, BC, Canada) was used to analyze the staining intensity, as reported before [30]. Values of IHC image analysis were expressed as mean ± SEM. According to the IHC staining density, the cases were divided into a high-expression group with a staining intensity above the average value and a low-expression group with a staining intensity lower than the average value.

### 4.16. TCGA and cBioPortal Database

We also analyzed the expression of the *LDHA* gene, *DOT1L* gene, and *BCAT1* gene using data obtained from TCGA (The Cancer Genome Atlas) through the cBioportal (https://www.cbioportal.org/, accessed on 1 January 2020) [31].

### 4.17. Statistical Analysis

Statistical analyses were performed using GraphPad Prism 7.0. All data shown represented results obtained from three independent experiments with standard errors of the mean (mean ± SEM). Progression-free survival (PFS) and overall survival (OS) curves were generated using the Kaplan–Meier method. Comparisons of the survival distribution were made with the log-rank test. Values of *p* < 0.05 were considered statistically significant. The symbols *, **, and *** denoted *p* < 0.05, *p* < 0.01, and *p* < 0.001, respectively, and ns meant non-significant.

## Data Availability

The datasets generated and analyzed in the present study are available from the corresponding author upon reasonable request.

## Supplementary Materials

The following supporting information can be downloaded at: https://www.mdpi.com/article/10.3390/ijms24119365/s1.

## Abbreviations

α-HB	α-hydroxybutyrate
α-KG	α-ketoglutarate
BCAAs	Branched-chain amino acids
BCAT1	Branched-chain amino acid transaminase 1
DOT1L	Disruptor of telomeric silencing 1-like
GSH	Glutathione
H_2_DCF-DA	Chloromethyl-2′, 7′-dichlorofluorescein diacetates
H3K79	Histone H3 lysine 79
IDH	Isocitrate dehydrogenase
LDHA	Lactate dehydrogenase A
OS	Overall survival time
GBM	Glioblastoma, *IDH* wild type
shRNA	Short hairpin RNA
siRNA	Small interfering RNA
ROS	Reactive oxygen species
TCGA	The Cancer Genome Atlas
TxN	Thioredoxin
