# Supplementary material for: Nuclear Translocation of LDHA Promotes the Catabolism of BCAAs to Sustain GBM Cell Proliferation through the TxN Antioxidant Pathway
Source: Int J Mol Sci. 2023 May 27;24(11):9365. doi: 10.3390/ijms24119365 (PMC10253380; doi:10.3390/ijms24119365)
Supplement: Supplementary file 1 [file ijms-24-09365-s001.zip › ijms-2367539-supplementary.pdf]

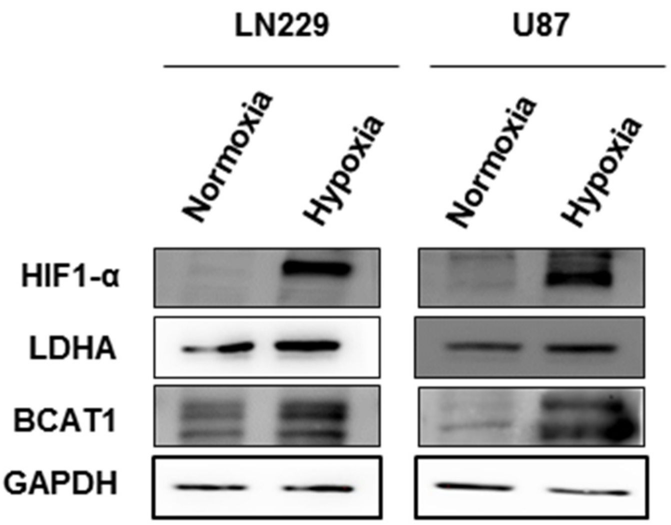

Figure S1. HIF-1α was induced after hypoxia culture, and the expression of LDHA and BCAT1 were increased.

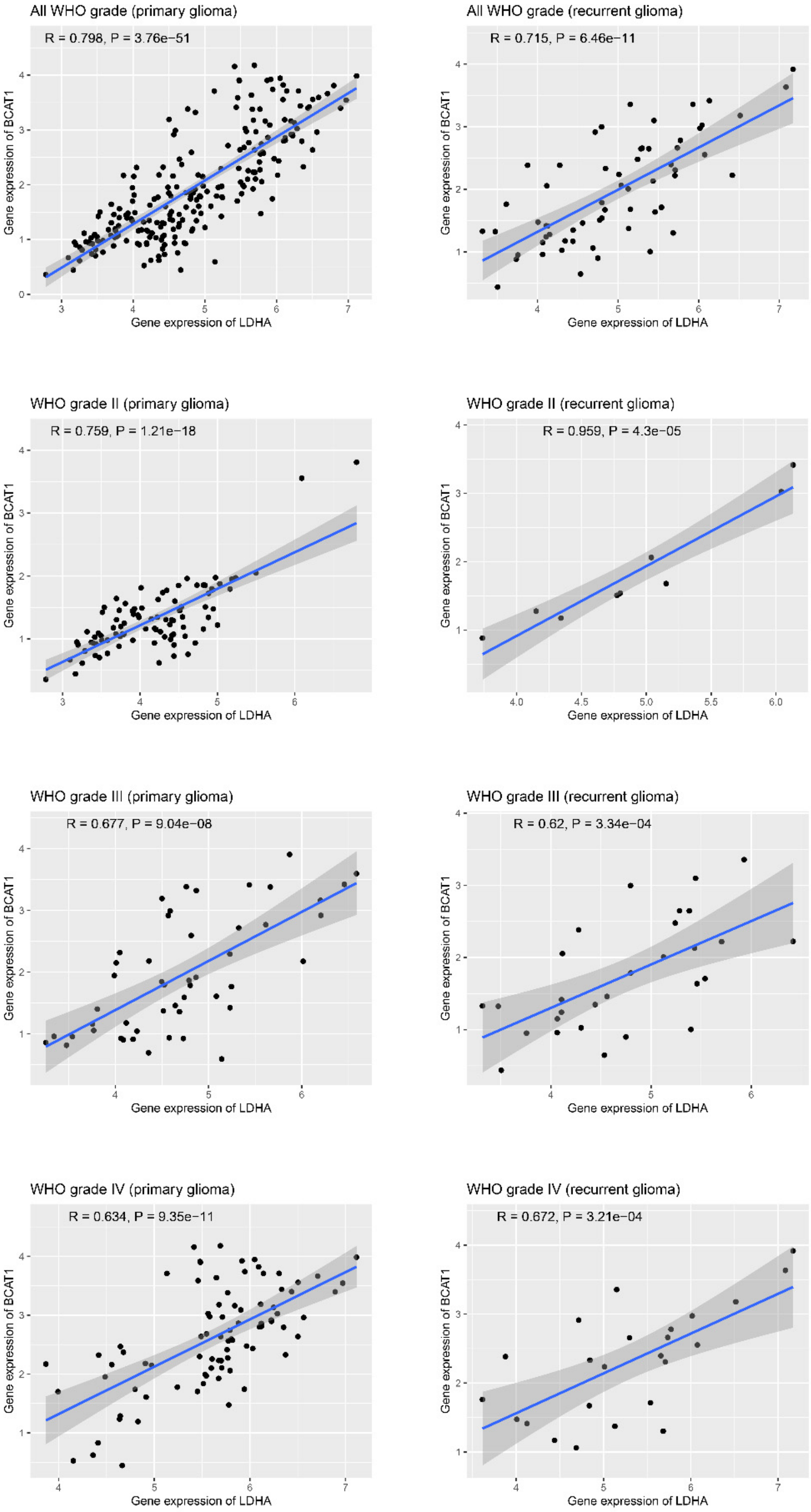

Figure S2. The correlation of LDHA and BCAT1 in glioma of CGGA. A significant positive correlation between LDHA and BCAT1 expression was found in both primary and recurrent gliomas, and a similar positive correlation was observed in gliomas at all malignant levels.
